# Strenuous Acute Exercise Induces Slow and Fast Twitch-Dependent NADPH Oxidase Expression in Rat Skeletal Muscle

**DOI:** 10.3390/antiox9010057

**Published:** 2020-01-08

**Authors:** Juliana Osório Alves, Leonardo Matta Pereira, Igor Cabral Coutinho do Rêgo Monteiro, Luiz Henrique Pontes dos Santos, Alex Soares Marreiros Ferraz, Adriano Cesar Carneiro Loureiro, Crystianne Calado Lima, José Henrique Leal-Cardoso, Denise Pires Carvalho, Rodrigo Soares Fortunato, Vânia Marilande Ceccatto

**Affiliations:** 1 Laboratório de Expressão Gênica, Instituto Superior de Ciências Biomédicas, Universidade Estadual do Ceará, Fortaleza 60.714-903, Brazil; juosorio@gmail.com (J.O.A.); luizhen-ricky@hotmail.com (L.H.P.d.S.); adrianoccloureiro@yahoo.com.br (A.C.C.L.); 2 Laboratório de Fisiologia e Sinalização redox, Instituto de Biofísica Carlos Chagas Filho, Universidade Federal do Rio de Janeiro, Rio de Janeiro 21941-902, Brazil; leonardo.matta@hotmail.com (L.M.P.); igorccdrm@hotmail.com (I.C.C.d.R.M.); rodrigof@biof.ufrj.br (R.S.F.); 3 Instituto de Educação Física e Esportes, Universidade Federal do Ceará, Fortaleza 60455-760, Brazil; ferrazalex@hotmail.com; 4 Laboratório de Eletrofisiologia Instituto Superior de Ciências Biomédicas, Universidade Estadual do Ceará, Fortaleza 60.714-903, Brazil; crys.calado@gmail.com (C.C.L.); lealcard@gmail.com (J.H.L.-C.); 5 Laboratório de Fisiologia Endócrina Doris Rosenthal, Instituto de Biofísica Carlos Chagas Filho, Universidade Federal do Rio de Janeiro, Rio de Janeiro 21941-902, Brazil; dencarv@gmail.com

**Keywords:** skeletal muscle fibers, reactive oxygen species, antioxidant defenses, oxidative metabolism fibers, glycolytic metabolism fibers

## Abstract

The enzymatic complex Nicotinamide Adenine Dinucleotide Phosphate (NADPH) oxidase (NOx) may be the principal source of reactive oxygen species (ROS). The NOX2 and NOX4 isoforms are tissue-dependent and are differentially expressed in slow-twitch fibers (type I fibers) and fast-twitch fibers (type II fibers) of skeletal muscle, making them different markers of ROS metabolism induced by physical exercise. The aim of this study was to investigate NOx signaling, as a non-adaptive and non-cumulative response, in the predominant fiber types of rat skeletal muscles 24 h after one strenuous treadmill exercise session. The levels of mRNA, reduced glycogen, thiol content, NOx, superoxide dismutase, catalase, glutathione peroxidase activity, and *PPARGC1α* and *SLC2A4* gene expression were measured in the white gastrocnemius (WG) portion, the red gastrocnemius (RG) portion, and the soleus muscle (SOL). NOx activity showed higher values in the SOL muscle compared to the RG and WG portions. The same was true of the NOX2 and NOX4 mRNA levels, antioxidant enzymatic activities, glycogen content. Twenty-four hours after the strenuous exercise session, NOx expression increased in slow-twitch oxidative fibers. The acute strenuous exercise condition showed an attenuation of oxidative stress and an upregulation of antioxidant activity through *PPARGC1α* gene activity, antioxidant defense adaptations, and differential gene expression according to the predominant fiber type. The most prominent location of detoxification (indicated by NOX4 activation) in the slow-twitch oxidative SOL muscle was the mitochondria, while the fast-twitch oxidative RG portion showed a more cytosolic location. Glycolytic metabolism in the WG portion suggested possible NOX2/NOX4 non-regulation, indicating other possible ROS regulation pathways.

## 1. Introduction

Physical exercise activity increases by approximately 20 times the volume of total oxygen consumed (VO_2_) and by 100 times the VO_2_ in the active muscle fibers, generating reactive oxygen species (ROS) in skeletal muscle fibers [1]. The contribution of the mitochondria (and no other sources) to the acute increase in ROS during muscular contractions was postulated by Sakellariou et al. [2], who showed that Nicotinamide Adenine Dinucleotide Phosphate (NADPH) oxidase complex effects are predominant above mitochondrial function. In this way, the NADPH oxidase—and not mitochondria—is the principal source of superoxide generation during short-term contractile activity.

The NADPH oxidase produces superoxide by intracellular NADPH electron transfer through the membrane, docking them to molecular oxygen, which leads to ROS formation [3]. These enzymes present isoforms, which can be differentiated by local expression and by the cofactors used for their activation [4].

In general, the NADPH oxidase contains six sub-units. Enzyme catalytic sites involve two transmembrane proteins (p22phox and p91phox), forming a flavocytochrome b558, three more cytosolic proteins (p47phox, p40phox and p67phox), and two 1 Rho guanosina trifosfatase GTPases (Rac1 and Rac2) [5]. Phagocytic cells express six proteins, denoted as NOX1, NOX3, NOX 4 and NOX 5 in addition to the NOX2 and NOX4 cited above, along with Dual Oxidase 1 and 2 (DUOX 1 and DUOX 2) and the GTPases Rac1 and Rac2 [5,6,7]

Skeletal muscles are composed of heterogeneous fibers from genetic, morphological and biochemical points of view [8]. The muscle classification of fiber types is related to the twitch type, which can be slow (type I fibers) and fast (type II fibers). Type II fibers are further subdivided into IIa, IIx and IIb [9]. The soleus (SOL) muscle is predominantly a type I muscle, the red gastrocnemius (RG) muscle is predominantly type IIa, and the white gastrocnemius (WG) is predominantly type IIb [10]. Muscular fibers have the capacity for phenotypic adaptation, the differential expression of isoforms, and the changing of muscle architecture in response to internal and external cellular environments [11].

Marked differences after three weeks of training were demonstrated between predominant slow-twitch type I and fast-twitch type II skeletal muscles in experimental treadmill exercise in terms of ROS and NOX expression and enzyme activity as an adaptation to chronic exercise [12]. That study demonstrated significant differences in the mitochondrial metabolism of ROS in muscles that presented some predominant fiber types, with a mitochondrial release of H_2_O_2_ (including Picard et al. [13]) that was two-to-three times higher in the SOL muscle compared to the WG portion of muscle fibers. Strenuous-intensity one-session exercise may exceed the redox capacity of the organic system and promote ROS production [14]. Even a minor ROS production increase after a unique exercise session could show a regulatory rule of cellular regulation pathways to an oxidative phenotype of the skeletal muscle [15,16]. Following this evidence, we believe in the existence of a characteristic NOX expression pattern in the different skeletal muscle fiber subtypes. In this perspective, this study’s objective was to evaluate if NOX activity and expression in skeletal muscle are specific for each type of fiber, to discern the possible contribution of oxidative cell differential expression, and to discern aerobic acute conditions adaptation.

## 2. Materials and Methods

### 2.1. Ethical Aspects

The experimental procedures were in accordance with Brazilian law and were approved by the Ethics Committee on Animal Use of State University of Ceara (CEUA/UECE) (no. 5236655/2016). Sixteen 2-month-old male Wistar rats (220–250 g) were kept under standard conditions: 12/12 h light-dark cycle, controlled temperature (22–25 °C), and food and water ad libitum.

### 2.2. Physical Exercise Sessions

**Figure 1:**
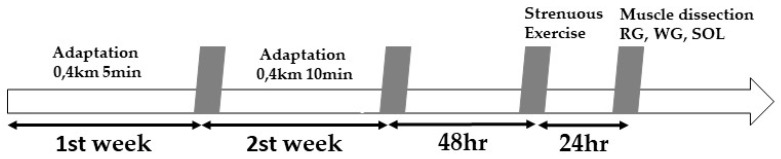
Experimental design.

All animals were initially acclimatized to a rodent treadmill (Inbramed—Porto Alegre, Rio Grande do Sul, Brazil) due to submit all subjects of study to the same environmental conditions by performing 10 adaptation sessions over two weeks in the nocturnal period. Acclimation was accomplished 5 days/week for 2 weeks: 1st week: 0.4 km/h for 5 min; 2nd week: 0.4 km/h for 10 min. Control animals only went through the acclimation period. At 48 h after the acclimation period, one single strenuous exercise session was performed that consisted of 3 min running stages with a constant load at an initial velocity of 0.3 km/h, with 0.2 km/h rises between stages, until the animals reached physical exhaustion (Figure 1), which was determined by the animal’s refusal to continue and loss of limb coordination [17,18,19].

Animals were anaesthetized with ketamine 60 mg/kg and xylazine 8 mg/kg, followed by decapitation at 24 h after the exhaustive exercise session. The soleus and gastrocnemius muscles were extracted and stored at −80 °C until further analyses. Before freezing, the gastrocnemius muscle was separated into red and white portions. 

### 2.3. Lactate Analysis

A total of 25 µL of blood was collected from the tail on the first and last adaption day on the treadmill, before and after the exhaustive exercise season. Immediately after collection, the sample of blood was transferred to a tube with 50 µL of sodium chloride, and this sample was read in a YSI 2300 Stat Analyzer (Yellow Springs Instruments, Yellow Springs, Greene County, OH, USA) [20].

### 2.4. Evaluation of Glycogen Content

Muscle tissue (100 mg) was digested in a KOH 30% solution and melted for 30 min. Analyses were performed following Dubois et al. [21]. Total sugar contents were determined by spectrometry at a wavelength of 490 nm by using a 1% glucose standard curve in a break of 10–90 mg.

### 2.5. Measurement of Reduced Thiol Residues of Total Protein

Reduced thiol content was measured with 5,5-dithioidobenzoic acid (DTNB) as a mediator. DTNB reacts with thiol groups that have been cleaved from disulfide bonds, and 2-nitro-5-thio benzoate (NTB) is formed as a product that ionizes to NTB^2−^ in alkaline water. NTB^2−^ was quantified by absorbance at 412 nm [22], and the results are expressed as nanomoles of DTNB reduced per milligram of protein.

### 2.6. Gene Expression by Quantitative PCR

Total RNA was extracted from muscle tissue by using a TRIzol reagent (Invitrogen, Waltham, MA, USA). RNA concentration and purity were determined with a spectrophotometer (Biomate 3S, Thermo Scientific, Waltham, MA, USA, at 260 and 280 nm. complementary DNA (cDNA) was synthetized from 2 µg of RNA in a thermocycler (Techne TC-412, Staffordshire, ST15 0SA, UK) with an ImProm-II reverse transcription kit (Promega, São Paulo, Brazil) according to standard methods. qPCR was performed with a Bio-Rad CFX96 System with gene primers for SOD 1 (superoxide dismutase 1, National Center for Biotechnology Information (NCBI) ID 24786), SOD 2 (superoxide dismutase 2, NCBI ID 20656), CAT (catalase, NCBI ID 24248), GPX1 (glutathione peroxidase 1, NCBI ID 24404), TBP (TATA box binding protein, NCBI ID 117526), and PGC1α (peroxisome proliferator-activated receptor gamma, coactivator 1 alpha, NCBI ID 83516) genes. Two microliters of cDNA were amplified by qPCR with 10 μL of Power SYBR Green PCR Master Mix (Life Technologies, Waltham, MA, USA), 300 nM of each reverse and forward primer, and 6.8 μL of ultra-pure water. The amplification parameters were 95 °C/10 min, 40 cycles of 95 °C/15 s, and 60 °C/1 min, and a gradual rise in temperature from 55 to 95 °C. Relative gene expression was determined by the 2-delta delta threshold cycle (2^−ΔCT^) method by using TBP as a constitutive gene. Fold change data are reported as arbitrary units.

### 2.7. Antioxidant Enzyme Activity

SOD activity was measured through adrenaline auto-oxidation inhibition assays, with absorbance read at 480 nm [23]. CAT activity was evaluated through the H_2_O_2_ decrease rate at 240 nm [24], and the results are expressed as U/total protein mg. GPX enzyme activity was assayed by NADPH oxidation in the presence of H_2_O_2_ and was read at 340 nm [25]. Enzyme activity readings were performed with a spectrophotometer (Biomate 3S, Thermo Scientific—Waltham, MA, USA), and the results are expressed as nmol/min/total protein mg. Protein concentration was determined by the Bradford assay [26].

### 2.8. NADPH Oxidase Activity

NADPH oxidase activity was quantified in the skeletal muscle by the Amplex Red/Horseradish Peroxidase Assay (Molecular Probes, Invitrogen, Waltham, MA, USA). Muscle tissues were homogenized in a 50 mM sodium phosphate buffer, pH 7.2, containing 0.25 M sucrose, 0.5 mM Dithiothreitol (DTT), 1 mM Ethylenediaminetetraacetic acid disodium salt dihydrate (EGTA), 5 mg/mL of aprotinin and 34.8 mg/mL of phenylmethanesulfonyl fluoride (PMSF). To obtain the microsomal fraction, the homogenates from muscle samples were centrifuged at 3000× *g* for 15 min at 4 °C. The supernatant was centrifuged at 100,000× *g* for 35 min at 4 °C, and the P100,000× *g* pellet was suspended in 0.5 mL of 50 mM sodium phosphate buffer, pH 7.2, containing 0.25 M sucrose, 2 mM MgCl_2_, 5 mg/mL of aprotinin, and 34.8 mg/mL of PMSF, and stored at −80 °C until H_2_O_2_ generation measurements. The microsomal fraction was incubated in a 150 mM sodium phosphate buffer (pH 7.4) containing superoxide dismutase (SOD) (100 U/mL; Sigma, St. Louis, MO, USA), horseradish peroxidase (HRP) (0.5 U/mL, Roche, Indianapolis, IN, USA), and Amplex Red (50 μM; Molecular Probes, Eugene, OR, USA), and the fluorescence was immediately measured in a microplate reader (Victor X4; PerkinElmer, Norwalk, CT, USA) at 30 °C by using an excitation wavelength of 530 nm and an emission wavelength of 595 nm. Specific NADPH oxidase activity was calculated by the differences between the activities in the presence and absence of NADPH. The specific enzymatic activity is expressed as nanomoles H_2_O_2_ per hour per milligram of protein (nmol·h^−1^·mg^−1^) [27]. Protein concentration was determined by the Bradford assay [26].

### 2.9. Statistical Analyses

The results were evaluated as the mean values from biological replicates and standard deviations (SD). Statistical significance was set at *p* < 0.05. For comparisons within groups, one-way and two-way ANOVA followed by Bonferroni’s multiple comparison test were used.

## 3. Results

### 3.1. Strenuous One-Session Exercise

**Figure 2:**
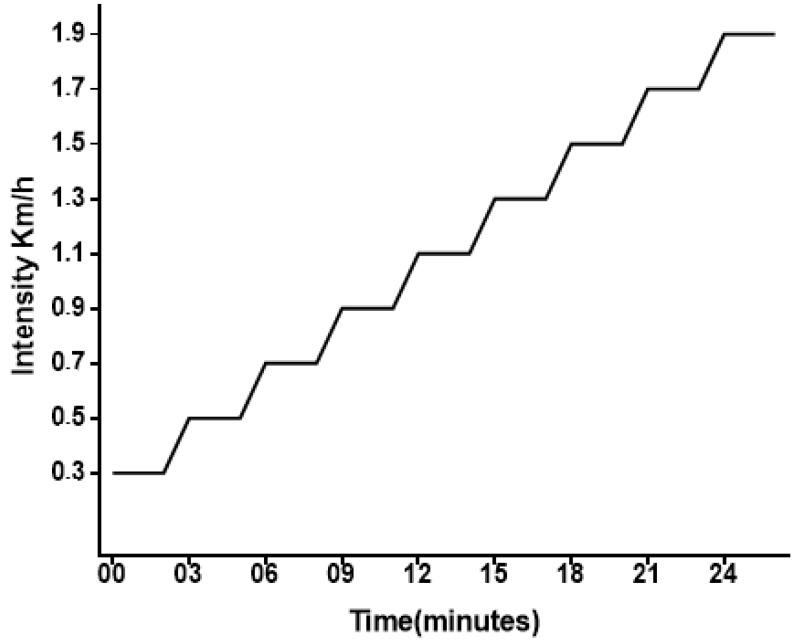
Schematic representation of the incremental load exercise.

This exercise session characterized the maximum individual exercise capacity according to the model proposed in Figure 2. Final exhaustion velocity and final time to exhaustion were measured as the absolute parameters of intensity and volume, equaling 1.9 ± 0.16 km/h and 24.92 ± 2.5 min, respectively. The exercise intensity parameters obtained were a power volume of 1.17 ± 0.13 W and physical work of 981.94 ± 163.45 J.

### 3.2. Biochemical Characterization

#### 3.2.1. Lactate Analysis

**Figure 3:**
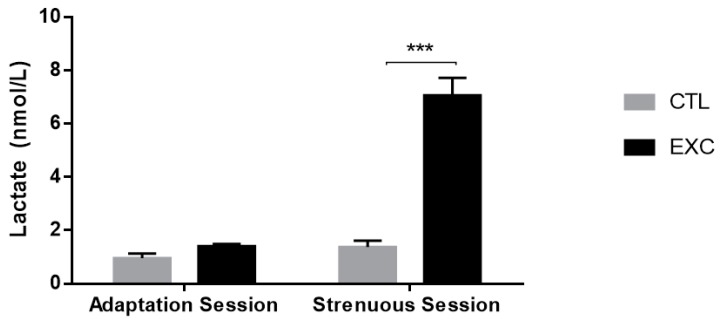
Lactate levels from the Control (CTL) and exercise (EXC) groups. Data are shown as mean ± S.E.M. (*n* = 8/group). *** *p* < 0.001.

The lactate levels before and after the adaptation session were not significantly different. The lactate level before the strenuous session was similar to the pre- and post-adaptation sessions, and the lactate level at the exhaustion point was higher in the exercised group when compared to its control (Figure 3).

#### 3.2.2. Muscle Glycogen Content 

**Figure 4:**
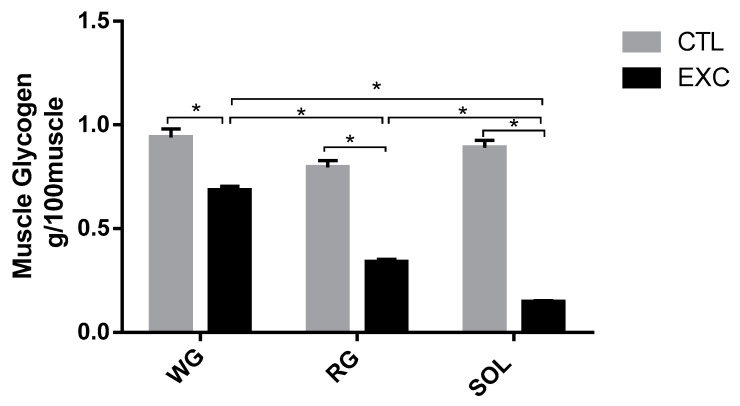
Glycogen content (mg/100mg tissue) in the white gastrocnemius, the red gastrocnemius and the soleus from the Control (CTL) and exercise (EXC) groups. Data are shown as mean ± S.E.M. (*n* = 8/group). * *p* < 0.05.

There was a significant reduction in muscle glycogen levels after strenuous exercise in different types of muscle predominant fibers. Moreover, there was a considerable reduction in glycogen in the SOL muscle (slow-twitch fibers) compared to fast-twitch fibers (RG and WG portions) (Figure 4).

### 3.3. Thiol Content

**Figure 5:**
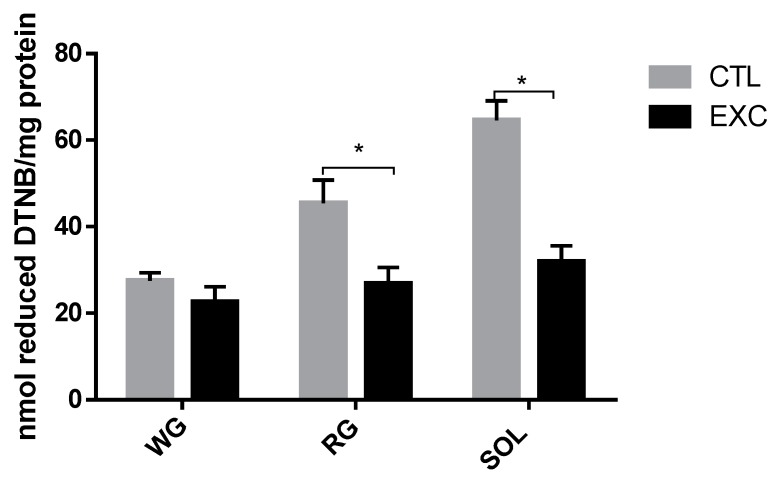
Reduced thiol content of rat skeletal muscles. Total sulfhydryl groups were measured by the reaction of thiols with 5,5-dithioidobenzoic acid (DTNB), evaluated in a spectrophotometer at 412 nm. Data are shown as mean ± S.E.M. (*n* = 8/group). * *p* < 0.05.

The results indicated a significant increase in the thiol group in the SOL muscle in the exercised group. The reduced thiol level in the RG and WG muscle portions did not show changes between groups. When comparing the different fiber types, it was observed that the thiol level was significantly higher in the SOL muscle, which presented higher NOx activity (Figure 5).

### 3.4. Effect of Acute Exhaustive Physical Exercise on NOX Activity and mRNA Levels in Different Types of Muscle Fibers

**Figure 6:**
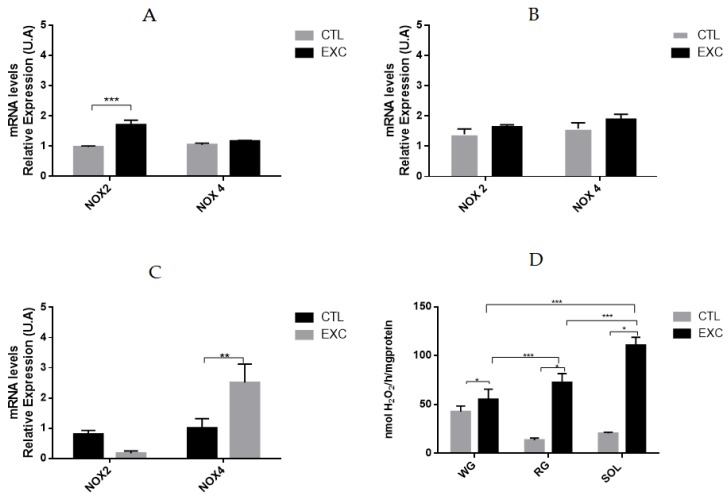
mRNA levels of the rat white gastrocnemius(**A**), red gastrocnemius (**B**), the soleus (**C**) from the Control (CTL) and exercise (EXC) groups. Nicotinamide Adenine Dinucleotide Phosphate (NADPH) oxidase activity (**D**). H_2_O_2_ production was determined in the microsomal fraction by the Amplex Red/Horseradish Peroxidase assay. mRNA levels were determined by qPCR and are expressed relative to the white gastrocnemius, the red gastrocnemius and the soleus muscle. Data are shown as mean ± S.E.M. (*n* = 8/group). * *p* < 0.05; ** *p* < 0.01; *** *p* < 0.001.

NADPH oxidase activity was significantly higher in the slow-twitch glycolytic SOL muscle compared to the slow-twitch oxidative RG and the fast-twitch glycolytic WG (Figure 6A). To better characterize the NOx isoforms responsible for the higher H_2_O_2_ generation in oxidative muscle fibers, we evaluated the mRNA expression of the NOX enzymes. After exercise, there were no differences in the expression levels of NOX2 or NOX4 mRNA in the fast-twitch white glycolytic gastrocnemius compared to the control (Figure 6B). However, the NOX2 mRNA levels increased in the fast-twitch oxidative red gastrocnemius muscle of the exercised group, while NOX4 mRNA levels remained unchanged (Figure 6C). In the slow-acting oxidative SOL, NOX4 mRNA levels were higher in the exercised group compared to the control group, while the NOX2 mRNA levels remained unchanged (Figure 6D).

### 3.5. Antioxidant Enzyme mRNA Levels in Different Types of Muscle Fibers

**Figure 7:**
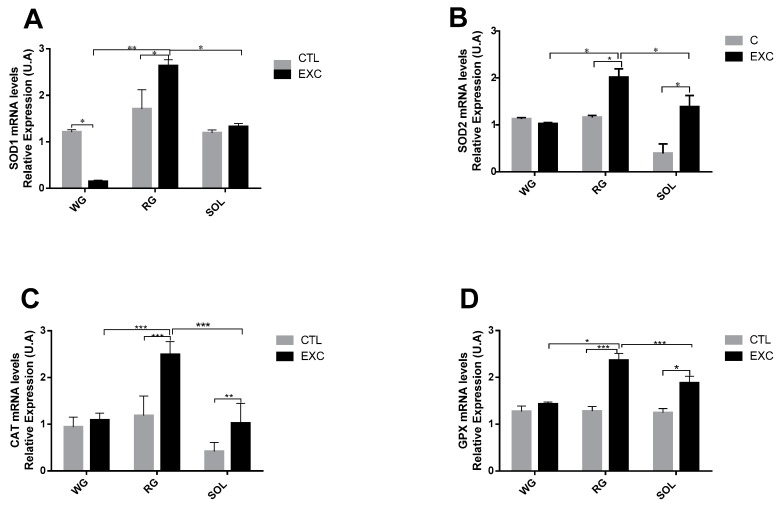
Quantification of mRNA expression of NADPH oxidase (NOX)-regulated antioxidant genes including superoxide dismutase-1 (SOD1) (**A**), superoxide dismutase 1 SOD2 (**B**), catalase (**C**) and glutathione peroxidase (GPX) (**D**) from the Control (CTL) and exercise (EXC) groups. Data are shown as mean ± S.E.M. (*n* = 8/group). * *p* < 0.05; ** *p* < 0.01; *** *p* < 0.001.

The results indicated a significant increase in SOD1 in the exercise group in the red portion of the gastrocnemius and a significant reduction in the white portion. There were no changes in the soleus in the exercise group. The SOD1 mRNA level was significantly higher in the RG muscle compared to the SOL and WG muscles (Figure 7A). In relation to the levels of SOD2 mRNA (Figure 7B), CAT (Figure 7C) and GPX1 (Figure 7D), we observed an increase in the exercised group compared to the control group in the red portion of the gastrocnemius and the soleus. No changes were observed in the exercise group in the white portion of the gastrocnemius. When comparing the different types of fiber, there was a higher expression of both enzymes in the RG muscle fibers compared to the WG and the SOL.

### 3.6. Antioxidant Enzyme Activity in Different Types of Muscle Fibers

**Figure 8:**
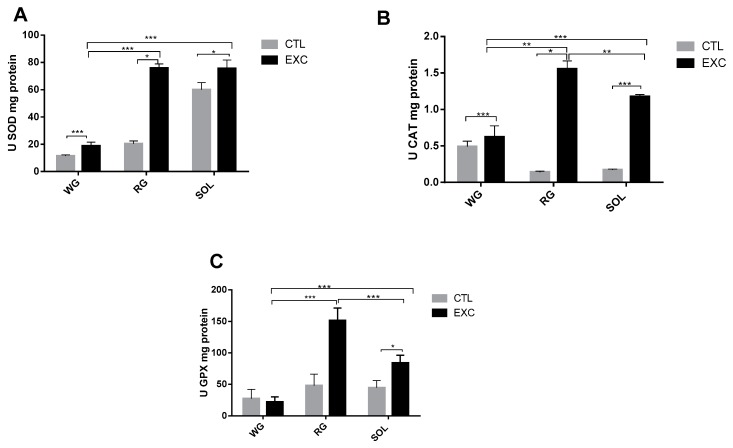
Enzyme activity SOD (**A**), CAT (**B**) and GPX (**C**) were measured by spectrophotometry and are expressed relative to the white gastrocnemius, the red gastrocnemius and the soleus muscle from the Control (CTL) and exercise (EXC) groups. Data are shown as mean ± S.E.M. (*n* = 8/group). * *p* < 0.05; ** *p* < 0.01; *** *p* < 0.001.

Next, the activity of the antioxidant enzymes SOD, CAT and GPX was analyzed by comparing the exercise group with the control group (Figure 8). The results indicated a significant increase of SOD activity and a significant reduction in the WG portion in the RG portion of the exercise group. There were no changes in the soleus exercise group. When comparing the different types of fibers, total SOD activity was significantly higher in the RG compared to the SOL and WG muscles (Figure 8A). In relation to the activities of catalase (Figure 8B) and GPX (Figure 8C), we observed an increase in catalase activity in the exercised group compared to the control group in the RG portion and the SOL muscle. No changes were observed in the WG. When comparing the different predominant fiber types, there was greater activity of both enzymes in the RG muscle compared to the WG and the SOL. The mRNA levels of the antioxidant enzymes were in agreement with the levels of enzymatic activity (Figure 8A–C).

### 3.7. PPARGC1α Redox Control and SLC2A4 mRNA Levels

**Figure 9:**
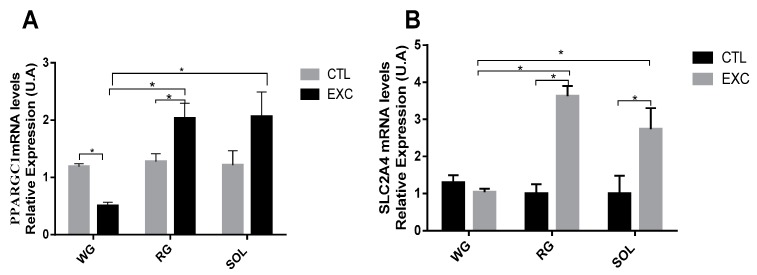
mRNA levels of *PPARGC1α* (**A**) and *SLC2A4*α (**B**) were determined by qPCR and are expressed relative to the white gastrocnemius, the red gastrocnemius and the soleus. Data are shown as mean ± S.E.M. (*n* = 8/group). * *p* < 0.05.

The mRNA level of the *PPARGC1α* gene increased in the exercise group compared to the control group in the RG portion and the SOL muscle, whereas it was reduced in the WG (*p* < 0.05). Furthermore, *PPARGC1α* gene expression was higher in the RG portion and the SOL muscle (*p* < 0.05) compared to the WG portion (Figure 9A). The mRNA level of *SLC2A4α* increased in the exercise group compared to the control group in the RG portion and the SOL muscle (*p* < 0.05) (Figure 9B).

## 4. Discussion 

Physical exercise has a positive physiological impact on antioxidant defense alterations [28,29,30]. Physical exercise prevents muscular atrophy [3], attenuates potential glycolytic metabolism alterations, and increases oxidative capacity in skeletal muscles in diabetic rats [19]. Mitochondrial alterations are associated with oxidative stress induced by skeletal muscles [31,32]. The NADPH oxidase has been linked to strenuous exercise in the modulation of the ROS generator system [2]. Previously, NOX activity and expression have differed according to the skeletal muscle’s predominant fiber type [12].

This premise is supported by the current findings on strenuous one-session exercise and skeletal muscle fiber type, suggesting the existence of different forms of NOX activation in muscle fibers related to antioxidant defense, glucose metabolism, and mitochondria. These characteristics were evaluated by qPCR the expression of oxidative stress marker genes, with a later analysis of oxidant and antioxidant activity, in order to obtain a better characterization of the studied skeletal muscle fibers. Results corroborate with the identified differential gene expression.

### 4.1. Lactate Accumulation and Glycogen Decrease

The occurrence of fatigue coincides with the accumulation of lactate and the depletion of muscular glycogen stores [33]. The lactate level rose during the exhaustive exercise session in the rats of the present study, indicating a greater effort compared to that of themselves in the first week of treadmill adaptation (Figure 3).

The muscle glycogen depletion observed in our study provides strong evidence that the exercise protocol caused the recruitment of all types of fibers (Figure 4). Moreover, the results suggest that there was a selective depletion of muscle glycogen, that is, most of the glycogen use occurred in slow-twitch fibers, which may indicate a greater recruitment of type I fibers in this type of exercise. Such an effect has been well established in the literature [34,35,36]. 

These data suggest an association between the reduction of intramuscular glycogen stores and the increase in reactive oxygen species in muscular fatigue. Though the mechanism by which the production of reactive oxygen species is accelerated during intense contractions, it is not yet fully understood, and a possible cause-and-effect relationship supports this association [37].

### 4.2. Thiol Content

The main non-enzymatic antioxidant pathway in muscle is the glutathione system, which employs thiol cycles to form an oxidant species matrix. Thiol is an organic molecule class with sulfhydryl groups that has important role in redox homeostasis through oxidation or reduction reactions. Beyond these protection mechanisms, ROS production levels can exceed myofibers’ antioxidant capacities, leading to oxidative stress and reducing muscle contraction forces [38]. The thiol levels in this study decreased after a strenuous session of exercise in predominantly slow-twitch oxidative tissue (SOL muscle) and fast-twitch oxidative tissue (RG portion), indicating an oxidative state (Figure 5). This function is especially important during strenuous muscle activity, when ROS production is substantially increased as part of the fatigue process [39].

### 4.3. Antioxidant Capacity and Mitochondrial Function

Some studies have demonstrated the presence of marked differences between slow-twitch fibers in ROS metabolism—differences caused by possible changes in metabolic regulation of calcium transition due to altered pore permeability [40,41,42]. These results suggest that specific mitochondrial phenotypes exist in slow- and fast-twitch fibers in skeletal muscle and are essential for muscular function. In this case, elevated levels of NOX activity in general were obtained because of the increased mRNA levels of NOX2 and NOX4 in the RG portion and the SOL muscle.

Strenuous one-session exercise was capable of significantly increasing the NOx activity in the RG portion and the SOL muscle, without differences in the WG portion (Figure 6). The cytosolic SOD1 and mitochondrial SOD2 isoforms and the CAT and GPX enzymes are molecular components of a complex antioxidant system. SOD is a first line of defense against the increase in mitochondrial superoxide or other sources of ROS, such as NADPH oxidase [43,44]. Previous studies have demonstrated that SOD level and activity could be increased in skeletal muscles without the significant alteration of their mRNA [44,45,46].

Skeletal muscle is a heterogeneous tissue in terms of oxidative capacity and protein turnover [44], presenting intense variations depending on fiber type predominance. In rats, SOD presents increased activity, in predominantly oxidative skeletal muscles, with a higher content of type I and IIa fiber types in comparison to fibers with lower mitochondrial content, i.e., IIx and IIb fibers [47,48]. Our data from a single strenuous exercise session support these results in the WG and RG portions and the SOL muscle. In addition to these differences in the antioxidant response to strenuous physical exercise with distinct phenotypical predominance, our results indicate that exercise could promote general SOD activity as oxidative capacity increases, such as in the RG portion. The mRNA content was consistent with the enzyme expression, except for SOD1 in the WG portion (Figure 7).

CAT and GPX have fundamental control over the H_2_O_2_ level in the redox system. However, physical exercise is associated with an increase in antioxidant defense in skeletal muscular tissues, with controversial results under acute exercise: Expression increases [49], decreases [50,51], and no alteration [50] have been observed. In our study, CAT activity and expression were increased, possibly by new H_2_O_2_ generation by strenuous exercise, in a tissue-dependent manner. In fact, the activity of CAT increased in the RG and WG portions and the SOL muscle (Figure 8), in line with the reported elevated CAT activity in oxidative fibers and reduced activity in others [52,53,54]. The new input could be obtained by several intracellular metabolic pathways, such as AMP-activated protein kinase(AMPK)/Peroxisome proliferator-activated receptor gamma coactivator 1-alpha (PGC-1α) as seen below.

GPX is one of the most important enzymes responsible for H_2_O_2_ cytoplasmic [55] and mitochondrial detoxification [13], and it is expressed in skeletal muscle, notably in highly oxidative fibers [50]. Like that of SOD, the influence of GPX varies with the predominant fiber type and is more pronounced in oxidative fibers. This study confirms this premise with its finding of increased GPX mRNA in the RG and the SOL, showing slow- and fast-twitch dependency, with the exception of the WG portion, which showed no alteration.

### 4.4. PGC-1α Regulation

Muscular oxidative activity showed elevated levels of the transcription factor *PPARGC1α* mRNA in this study (Figure 9). This result suggests an intracellular protein increase with post-transcriptional activation mediated by AMPK and p38 Mitogen Activated Protein Kinases (MAPK) phosphorylation. PGC-1α acts by coactivating other transcription factors, including coactivators that are capable of histone acetylation. This coactivation is involved in transcriptional regulation and RNA processing [56]. 

PGC-1α coactivates many genes that are linked to energy metabolism, such as the *SLC2A4* gene, which encodes a principal glucose transporter in skeletal muscles [54]. Previous studies have supported this up- and downregulation in cell culture [57] in *PPARGC1α*-deficient mice and in *PPARGC1α*-overexpressing mice [58]. Thus, the predominantly oxidative RG and SOL showed a strong transcriptional response of the *SLC2A4* gene. Strenuous one-session exercise induced a high response in the genetic regulation of intracellular energy metabolism in oxidative tissues.

Preliminary studies have shown the relationship between antioxidant proteins such as SOD1, SOD2 and GPX (revised by Kang; Ji, [56]). Our study has shown that RG muscle, with increased antioxidant defense (SOD2, CAT, and GPX), is predominantly fast-twitch oxidative in a tissue-dependent way. This is almost certainly a function of the cited induction of *PPARGC1α* gene expression. Though the differences in the antioxidant defense between tissues are still unclear, the distinct phenotypic patterns of predominantly oxidative or glycolytic must include H_2_O_2_ detoxification, which is potentialized by the antioxidant enzymes SOD, CAT and GPX. PGC-1α shows a response in its basal genetic regulation, and H_2_O_2_ levels increase, possibly due to RG antioxidant enzyme responses by fiber recruitment in this tissue [13].

### 4.5. NOx Isoforms and Effect on Oxidative Stress

This study suggests that NOx expression depends on the muscle fiber type. The results showed that NOX2 signaling modulated the exercise response in fast-twitch fibers and that NOX4 signaling did so in slow-twitch fibers. Mitochondrial detoxification (indicated by NOX4 activity) showed an increased activity in the slow-twitch oxidative SOL muscle, while in the fast-twitch oxidative RG portion, it showed a predominant cytosolic location. Glycolytic metabolism in the WG portion showed possible NOX2/NOX4 non-regulation, indicating other possible ROS regulation pathways.

Superoxide generation is associated with electron leakage and incomplete O_2_ metabolism by the mitochondrial electron transport chain, including mitochondrial complexes I and III but also, as recently discovered, complex II [59]. However, the present study suggests that NOx expression is the main modulator of cytosolic superoxide production in slow-twitch oxidative muscle fibers, where greater *PPARGC1α* expression also occurs, implying that responses in the antioxidant defense pathways may occur in a tissue-dependent manner and preferentially in more slow-twitch oxidative tissues. Despite the differences in the antioxidant defense response between tissues with distinct phenotypic predominance, it has been suggested that the H2O2-detoxification capacity [13] and the activity of antioxidant enzymes (SOD, CAT and GPX) [16] are considerably lower in less oxidative muscle fibers. Only twenty-four hours waiting after a strenuous exercise session perhaps led to the finding of non-cumulative ROS stress response.

These differences involving different slow- and fast-twitch-predominant fiber types may be associated with *PPARGC1α* expression levels, since this coactivator regulates the gene expression of antioxidant enzymes, both in basal conditions and in response to increased H2O2 levels [13]. In the present study, the greater expression of *PPARGC1α* found in the more oxidative RG portion supports this hypothesis, suggesting that the impact of a strenuous session of exercise on the antioxidant response 24 h later in more oxidative fibers was possibly due to the higher recruitment of these fibers during exercise.

### 4.6. Potential Application

Our findings show that the ROS detoxification site in skeletal muscle is different according to the type of muscle fiber. In type 1 (slow) fibers, it was observed that mitochondria are the fundamental site in ROS detoxification. In type 2 (rapid) fibers, it was observed that detoxification occurs at a cytoplasmic level.

Evidence supports that endurance training induces differentiation and adaptation to type 1 (oxidative) fibers, and these fibers are characterized by having more mitochondria. Long-distance runners, whose effort is related to higher training volume, have an increased respiration rate, ATP production and mitochondrial antioxidant defense [60]. For these runners, increasing exercise intensity seems to be related to increased mitochondrial antioxidant defense due to ROS-mediated detoxification and signaling.

Sprint training, whose effort is related to higher training intensity and short running distances, has a higher activity of glycolytic pathway-related enzymes. Short distance runners have a higher content of type 2 (fast) fibers, and these fibers are characterized by more glycolytic capacity [61]. For these runners, increasing exercise intensity seems to be related to higher ROS production due to muscle contraction. Endogenously-produced ROS plays an important role in the changes in the glycolytic activity of Glucose *transporter* type 4 (GLUT4), hexokinase and phosphofructokinase (enzymes involved in glycolysis) [62].

In both conditions, increased exercise intensity resulted in increased ROS in response to muscle contraction. This increase may have been related to factors such as the amplification of intracellular signaling (cytosolic and nuclear), increased enzyme activity, increased antioxidant defense, increased contractile capacity and muscle endurance. These factors are critical to increased performance.

## 5. Conclusions

Twenty-four hours after one strenuous session of exercise, NOx expression in skeletal muscle was found to change according to the predominant muscle fiber type, with increased values in slow-twitch oxidative fibers. In addition, the results indicate that exercise attenuated redox imbalances and up-regulated antioxidant activity through *PPARGC1α*. Antioxidant defense adaptations and gene expression mediated by acute exhaustive exercise can occur differently according to the tissue’s predominant metabolic type.

The most prominent location of detoxification (indicated by NOX4 activation) in the slow-twitch oxidative SOL muscle was the mitochondria, while in the fast-twitch oxidative RG, the detoxification showed a more cytosolic location. Glycolytic metabolism in the WG portion showed possible NOX2 and NOX4 non-regulation, indicating possible other ROS regulation pathways.
